# A new definition of burnout syndrome based on Farber's proposal

**DOI:** 10.1186/1745-6673-4-31

**Published:** 2009-11-30

**Authors:** Jesús Montero-Marín, Javier García-Campayo, Domingo Mosquera Mera, Yolanda López del Hoyo

**Affiliations:** 1 Department of Psychiatry, Miguel Servet University Hospital, University of Zaragoza, Spain; 2 Spanish National University of Distance Education, Huesca, Spain; 3 Department of Psychology, University of Zaragoza, Spain

## Abstract

**Background:**

Although diverse definitions have been construed for burnout syndrome, most authors consider it to be a single phenomenon, the result of chronic work-related stress. However, in order to enable specific intervention strategies to be adopted, it is first necessary to establish different profiles for the syndrome. In this respect, have been proposed three burnout types ("frenetic", "underchallenged" and "worn-out"), each of which requires different means of dealing with frustration in the workplace. This study is an attempt to define and systematize the properties that characterize this typology proposal.

**Methods:**

For this purpose, the documents considering preliminary typology were examined by means of qualitative content analysis supported by grounded theory. Semiotic analysis was then performed on the core category resulting from the previous analysis.

**Results:**

A classification criterion, made up of three different burnout subtypes ("frenetic", "underchallenged", and "worn-out") capable of integrating the entire proposal was formulated.

**Discussion:**

Understanding the development of burnout syndrome, as a succession of stages characterized by the progressive diminishing of dedication to work, could serve for the establishment of specific therapies and for the prevention of the syndrome.

## Background

Burnout syndrome is considered an important work-related illness in welfare societies. It was through observations by Freudenberger [1] inside a detoxification clinic in the mid 1960s that the first scientific descriptions came to light of staff affected by this disorder. It was only in the 1980s that evaluation criteria for the syndrome became available, through the design of a standard measurement instrument, the Maslach Burnout Inventory or MBI [2]. Burnout is a psychosocial syndrome. It involves feelings of emotional exhaustion, depersonalization and diminished personal accomplishment at work. Emotional exhaustion is a situation where, owing to lack of energy, workers perceive they are no longer able to participate on an emotional level. Depersonalization entails the development of negative attitudes and feelings towards persons for whom work is done, to the point where they are blamed for the subject's own problems. Diminished personal accomplishment is a tendency in professionals to negatively value their own capacity to carry out tasks and to interact with persons for whom they are performed, and feeling unhappy or dissatisfied with the results obtained.

The MBI questionnaire has been adapted for application not only to human services professions but to all types of occupations in general. An updated definition of burnout, constructed using the latest version of the MBI [3], is that proposed by Maslach et al. [4]. In their description it is "a prolonged response to chronic emotional and interpersonal stressors on the job, and is defined by the three dimensions of exhaustion, cynicism, and inefficiency". Exhaustion is the feeling of not being able to offer any more of oneself at an emotional level; cynicism is contemplated as a distant attitude towards work, the people being served by it and among colleagues; ineffectiveness is the feeling of not performing tasks adequately and of being incompetent at work.

Burnout is generally considered a response by a subject to chronic work-related stress in an attempt to adapt or protect oneself from it [5]. From a transactional approach, stress is defined as "the result of a relationship with the environment that the person appraises as significant for his or her well-being and in which the demands tax or exceed available coping resources" [6]. This is the case because a life event is not what produces stress; rather, it is caused by the appraisal the affected person makes of it [7]. According to Lazarus and Folkman [6], coping is "cognitive and behavioural efforts to manage specific internal and/or external demands that are appraised as taxing or exceeding the resources of the person". A person will be psychologically vulnerable to a determined situation if he or she does not possess sufficient coping resources to handle it adequately, and if at the same time, he or she places considerable importance on the threat implicit in the consequences of this inadequate handling [6]. From this perspective, burnout syndrome may be seen as a progressively-developed process resulting from the use of the relatively ineffective coping strategies with which professionals try to protect themselves from work-related stress [5].

Burnout has also been described as an experience where the worker is aware of considerable discrepancy between his or her efforts and the results, between the invested efforts and the rewards obtained at work [8-14]. This phenomenological analysis framework is introduced into the subjective experience of those affected, and the conclusion is reached that the burnout process is triggered when the worker feels that his or her efforts are disproportionate to the gratification achieved, and consequently is no longer able to justify or cope with further investment of effort [10]. Burnout syndrome may be seen as the continuous perception that efforts made to carry out tasks are not effective, because expected gratitude, recognition or success at work are not being achieved [9,12].

Farber [14] criticizes the fact that most researchers have contemplated burnout as a single phenomenon, i.e. as a syndrome with relatively consistent aetiology and symptoms in all individuals. On the contrary, he proposed differentiation of the syndrome based on the description of three clinical profiles [8-14]. These different types of burnout, which the author classes as "frenetic", "underchallenged" and "worn-out", could be the result of different ways of responding to stress and frustration at work. The frenetic type works increasingly harder until he or she is exhausted and seeks satisfaction or success to equal the stress caused by the invested efforts. The underchallenged type is presented with insufficient motivation and must therefore cope with monotonous and unstimulating work conditions that do not provide necessary satisfaction. The worn-out type gives up when faced with too much stress or very little gratification at work. Consequently, while some professionals cope with dissatisfaction by investing greater effort in an attempt to achieve expected results, others cope by neglecting their tasks, in an attempt to balance the reasoning between rewards and their investment [11,12,14].

According to Farber [8] individual burnout treatments should be designed in relation to the aetiology and symptoms present in each subject. Thus, a level of specification in the treatment attending to individual differences would need to take into account the source of the feelings of frustration and clarify the stressors endured, the way of coping with them and the symptoms the syndrome is manifested through [13]. Farber's intuitive classification of burnout syndrome has raised the possibility of questioning the uniformity of the syndrome, and considers the need to design more specific therapeutic approaches. Nevertheless, in order to speak seriously of a typology, we need to look at a construct made up of abstract elements integrated into a unified conceptual model where there may be intensification of one or two aspects of concrete experience [15]. Farber's proposal for a typology does not achieve this degree of systematization, as it is not conceptually designed by means of abstract terms ordered over the same dimension.

The purpose of this research work is to resolve this lack of formal precision. Its principal aim is to explore and describe the attributes that could be used to characterize each of the clinical profiles proposed by Farber. Our secondary aim is to establish a classification criterion through which the generated conceptual structure would make sense, with the further intention of productively consolidating a new theoretical model.

## Method

We have adopted a qualitative social research approach and make use of the strategy known as documentary analysis [16]. The documentation covered by our analysis comprised the totality of the published writings of Barry Farber that impart his typological proposal. When selecting the corpus, we contacted the author in order to put together a list of all of his references. The selected texts comprised a total of seven written documents: three scientific articles, three book chapters and one communication [8-14]. Throughout his scientific output, the author highlights the experiences and interpretations of his own patients through a large number of direct quotes. Together with this, he has attempted to approach the object of his study from an existential perspective, which places his work on a level of humanistic strategy close to phenomenology in his version applied to clinical research.

Farber developed his theory model from his clinical observations of teachers, although he states that it is applicable to service professions in general. He also based his findings on the results of in-depth interviews with sixty psychotherapists (psychiatrists, psychologists and social workers, with different levels of experience and from both public and private practice). The results of this work are presented in one of the documents included in the textual corpus [8], although the article did not cite the psychotherapists directly, the author makes reference to them throughout his elaborate text. In other works [9,10,13], in addition to the author's explanations, we do find direct references to the interviewees (six primary and secondary school teachers, a number of them still active and others who finally chose to give up their profession, both male and female between twenty-six and fifty-six years of age, and with experience in education ranging between three and thirty years. Other works included in the corpus [11,12] provide a much more elaborate theory, while the last [14] is a preliminary validation study.

In order to reveal the levels of meaning underlying the surface of the corpus, we have made use of the methodological technique for obtaining information known as content analysis. According to Piñuel [17], content analysis is a series of procedures for the interpretation of communication products (messages, texts discourses) originating in unique, pre-recorded communication processes. Based on measuring techniques, at times quantitative (statistical techniques based on unit counts), at times qualitative (logical techniques based on a combination of categories), their purpose is to elaborate and process relevant data on the conditions under which those texts were produced, or on the conditions that may arise for their later use.

The type of content analysis used was of a qualitative, vertical and interpretative nature [18], with a projected sampling design and an emerging and non-frequential design for the analysis categories [16], all of which followed the analytical procedure provided by grounded theory. This procedure is a development on the phenomenological perspective, which becomes its intellectual root [16]. It is therefore congruent with the characteristics of the corpus. It is based on the "constant comparative method" [19], a strategy that enables concepts to be systematically generated and analysis and explicit coding to be combined with theory building. This type of analysis sets out to construct conceptual categories, marking their properties or significant features and the hypotheses that establish relations between all of them.

The following procedure was observed. A team of researchers comprising a native translator, two clinical psychologists and a psychiatrist worked together to achieve the translation of Farber's texts into Spanish and to divide the corpus into theme units using a structure of semantic fields [18]. Under mutual agreement, the research team subsequently made their first classification of the units, differentiating themes in general, which allowed them to separate references to typology from the other themes. By means of "open coding" [20], provisional interpretations of the segments belonging to the typology reference group were made. For this, the information contained in each of the selected units was compared and a common conceptual denomination was assigned to the group of segments sharing the same clinical profile as a standard.

As a next step, we set out to discover the properties of each of the profiles. We used a new type of classification, "axial coding" [21], consisting of intense analysis focused on one category each time. This new form of analysis, performed independently by each of the researchers, comprised an active and systematic search for properties by means of the constant comparison of the segments referring to each of the profiles separately. At the same time, interpretative notes were written down, which allowed relations to be established between the emerging properties. Finally, in order to define an agreed system that summarized the properties for each type, the characteristics obtained by each researcher were brought together and agreement was achieved on a total of five perfectly defined and mutually exclusive attributes for each profile.

The possible relations between the properties were clearly expressed in order to represent a highly parsimonious solution, which enabled the emerging conceptual structure to gain density. Once agreement was reached with regard to possible relations, we were able to reduce the theoretical framework by means of the merger and transformation of related properties into others on a higher level. Characteristics that belonged to disorders other than burnout, such as emotional disorders caused by anxiety or depression were also excluded. The result of this process gave rise to a total of nine sub-categories, three for each type, which summarized the properties of the entire typology.

At this point, we set out to develop a "core category" [21] that was able to express the totality of the typology coherently in a single dimension. For this purpose, we attempted to decide which of the properties best summarized the characteristics of each one of the profiles. Once the outstanding property for each profile was agreed on, we developed a category virtually able to integrate these three basic properties in one single dimension. By means of "selective coding" [21] of the corpus through the properties coming under this new core category, we observed how this category indeed provided the complete typology with an integrated, essential core framework, which was the nucleus of the emerging theory.

Once this stage was reached, we adopted a stance under the structuralist paradigm, making use of the semiotic square technique. For Floch [22], the semiotic square is a basic instrument of semiotic study and serves for the development of typologies. Abril [23] speaks of the semiotic square as a canonical representation of a set of relations. Quoting Greimas, Imbert [24] states that it is "the visual representation of the logical articulation of any semantic category...through which a description of the organizational model of signification is noted and its form of production by means of a typology of elemental relations". These relations are: contradiction, contrariness and the ability to be complementary, which are based on simple operations of assertion and negation, and by means of which the relation of reciprocal presupposition maintained by the primitive terms of the same semantic category are formalized. We used the end values of the core category as primitive terms for the analysis, and, by means of a review of their elemental relations, we formalized a classification criterion that finally gave meaning to the conceptual structure of the entire typology.

As can be appreciated, we have chosen a large combination of methodological triangulation perspectives, strategies and techniques, with the aim of increasing the consistency of the study. This was because we accepted the idea that qualitative research is inherently multi-method in focus [25]. Therefore, by consciously combining the elements referred to, we tried to give greater scope, rigour and depth to our study.

## Results

### a) Types Of Burnout

In Table 1 we describe the properties that characterize the clinical properties of burnout syndrome based on our study and according to the content of the analysed descriptions.

**Table 1:** Properties of three burnout types

FRENETIC	UNDERCHALLENGED	WORN-OUT
-Involvement in work.	-Indifference and superficiality in tasks.	-Neglecting responsibilities.
-Ambition and need for achievements.	-Lack of personal development.	-Absence of control over results.
-Inability to acknowledge failure.	-Contemplating another job.	-Problems with reward system.
-Neglecting own needs.	-Monotony and boredom.	-Difficulties in performing tasks.
-Anxiety and irritability.	-Absence of overload-induced stress.	-Depressive symptomatology.

### 1. Frenetic type

The frenetic type can be seen as a category of subjects who are highly applied and committed to their work, and who are greatly characterized by the investment of an enormous amount of time and effort in his or her dedication to work. These are subjects whose feelings of dissatisfaction cause them to increase their inputs, and are described by the author as

*"Those who in response to frustration work increasingly harder"*.

(Farber, 1990, p. 35)

#### 1.1. Involvement in work

A frequently described property of this profile is the increasing effort the subject makes when faced with his or her difficulties at work in an attempt to raise the probability of producing expected results. This characteristic has been conceptualized as involvement and is reflected in the corpus by Farber thus,

*"Those who in response to frustration work even harder in an attempt to produce the results they expect"*.

(Farber, 1990, p. 40)

The author cites the example of a frenetic individual (Paula, twenty-six years old, primary school teacher, two years' experience at work) who left her career with the feeling of not being able to give more of herself, probably because

"For the most part, she reacted to the strains at work by doubling her efforts..."

(Farber, 1991b, p. 119)

The frenetic type is a profile for tenacious and energetic persons, who cope with adversity with considerable enthusiasm and interest, doing all they can and giving all they are able to give. When they perceive that the results obtained do not correspond to the invested effort, they work with more determination to meet the goals they set initially. Farber explains that

"In the face of adversity and anticipated failure, these teachers often intensify their efforts and do everything possible to make classroom success more likely."

(Farber, 2000b, p. 682)

These subjects appear to believe that their efforts will lead them to success. They feel they are capable of overcoming all obstacles on their own and, consequently, they only need to reach the point where their investment will produce results. As reported by Farber,

"When input fails to achieve the hoped-for output (...)(they) work harder and harder in the belief that a point will be reached where their efforts finally will succeed."

(Farber, 2000b, p. 682)

#### 1.2. Ambition and need for achievements

Another of the properties characterizing the frenetic type is ambition, in the sense of the considerable need for achievements and external approval resulting from brilliant operations. This property is accompanied by great expectations in relation to performance, behind which we can presume there is a strong desire to feel special and gain admiration. This profile therefore attempts to surpass others by trying to be the best at his or her job. This was expressed by one of Farber's patients (Susan, thirty, high school teacher, three years' experience),

"Why do I always have to prove that I'm better than everyone else around me?"

(Farber, 2000b, p. 684)

Frenetic workers begin their careers with ambitious, sometimes unrealistic aspirations based on an idealistic view of the world. They seek good results without recognizing the negative aspects of their *modus operandi *and fantasize with the idea of accomplishing significant goals, placing themselves under growing pressure caused by their exaggerated need to obtain praise and distinction. As we can observe in the course of a psychotherapy session with Susan:

-*" (...)I kinda like thinking of myself as, well, maybe a little gutsier or more unflappable than most people*.

-*Unflappable?*

-*That I won't give up even when others would. That I give more than anyone else would and care more than anyone else*.

-*That makes you special and I guess that that feels good*.

-*Yeah, it does*.

-*I think we need to talk about why it's so important to feel special in this way (...)"*

(Farber, 2000b, p. 684)

Seduced by ideas of moral superiority, these subjects like to think that only they know how to properly solve matters related to their jobs, and experience satisfaction from the expectation that others will be able to discover their skill and sacrifice. They come to justify their action with altruistic arguments (they even feel guilty if they do not meet the objectives they set for themselves) and criticize people who do not share or understand their commitment and perfectionistic obsession. The author considered these ideas in the course of a psychotherapy session with Susan,

"...(we) began exploring the roots of her need to be perfect, better than others, and/or excessively admired by others for her apparent selflessness."

(Farber, 2000b, p. 683)

#### 1.3. Inability to acknowledge failure and difficult situations

Frenetic subjects are unable to accept failure or distinguish difficult-to-solve situations. They do not tolerate the limits set by reality owing to their strongly-instilled belief that the results of their work reflect personal worth and will. According to Farber,

"(...) the acknowledgement of failure is nearly impossible inasmuch as it reflects on their personal worth as human beings."

(Farber, 1990, p. 40)

Defeat is unthinkable for this profile of subjects as they understand work as an extension of themselves that must be successfully proven. Results to the contrary would damage their self-esteem given that it is based on the achievements reached and fulfilled expectations. Desperate to prove that they are capable of achieving what they set out to, these subjects strive endlessly in an effort to secure their personal worth. Therefore,

"(...) feeling so energetic and optimistic (or so desperate to prove themselves and regain some measure of self-esteem) that they invest more than ever and more than is healthy in their work (...) "

(Farber, 1991a, p. 97)

Although these results are at times imposed by the very nature of the problem, frenetic subjects fight daringly and desperately against all manner of odds and refuse to change their outlook so as not to compromise the integrity of their value system. According to the author,

"Individuals who fall in this category believe in maximum effort till success, with no let-up allowable; failure is never attributed to the nature of the problem but is always seem as a failure of will."

(Farber, 1991a, p. 90)

#### 1.4. Neglecting own needs

Frenetic individuals are so completely focused on obtaining results that they can even neglect their own needs, which means risking their health and personal life as they exert themselves without letting-up for long periods of time. They subject themselves to great pressure,

"These individuals risk their physical health and neglect their personal lives to maximize the probability of professional success."

(Farber, 1990, p. 40)

They suffer from the constant intrusion of their jobs into their private lives and feel they have failed to keep their work in perspective, given that they have not attained a balance between personal and professional needs. In Susan's words,

"I don't even have time to see my friends. I'm too tired or I'm busy planning."

(Farber, 2000b, p. 684)

These are excessively dedicated subjects, with an intense and incessant work pattern that determines a pattern of counterproductive efforts. They believe they can keep up their levels of exertion continually, until they are no longer able to cope and become exhausted or even ill, becoming emotionally and physically drained.

"They may appear to be frazzled or harried; nevertheless, they continue to work and attempt to solve problems at a nearly non-stop pace. Individuals rarely can sustain this energy indefinitely (although those suffering from classic burnout usually believe they can). They typically succumb to emotional and/or physical exhaustion."

(Farber, 2000b, p. 682)

Describing Paula's state before leaving her profession, Farber says:

"She felt she just could not keep up the pace of her efforts and was tired..."

(Farber, 1991b, p. 120)

#### 1.5. Anxiety and irritability

Continuous insistence under these conditions, in an attempt to satisfy their needs of achievement at the cost of overinvolvement and neglect of their own health, without acknowledging their own limitations, only increases the stress experienced by subjects of this type. Susan describes her situation this way:

"I really feel like I'm at the edge..."

(Farber, 2000b, p. 683)

This situation ends up exhausting internal resources and can lead to the development of clinical symptoms of anxiety owing to excessive worry about work demands. Subjects who have reached this stage have the sensation of feeling changed, altered and overwhelmed, and try to seek help by complaining of

"...anxiety, anger, confusion, teariness, and sleep problems..."

(Farber, 2000b, p. 681)

Stress ensuing from excessive exertion causes difficulties in resting or even sleeping. It leads subjects to enter a state of anxiety and irritability that produces continual anger and outbursts of rage directed at persons surrounding them. Referring to Susan, Farber says that

"She also expressed a great deal of anger toward her boyfriend for 'failing to understand' the importance of her work to her."

(Farber, 2000b, p. 682)

### 2. Underchallenged type

The underchallenged type is made up of subjects who have lost interest in their occupations and carry out their work tasks in a superficial manner. This is a group of subjects who cope with problems at work without too much involvement, seeing as they have lost their motivation along the way. In short, they are empty of challenges, motivation or desire for engagement.

"Those who perform their work perfunctorily, having lost interest in work they now find unchallenging"

(Farber, 1990, p. 35)

#### 2.1. Indifference and superficiality in tasks

An important property of this clinical profile is the indifference with which subjects cope with tasks. This is understood to be a way for them to perform tasks in a superficial and detached manner, although without reaching the point of neglecting their professional responsibilities altogether. Work is not appealing enough to justify greater investment of dedication, and the subject has partially lost interest in his or her commitments. According to Farber, the attitude expressed in the way of speaking of those affected is:

"...there's a job to do and I'll do it reasonably well, but I won't go out of my way to do it particularly well because the job isn't sufficiently engaging or interesting."

(Farber, 1990, p. 41)

These detached subjects cope with obstacles in their work by reducing their energy and enthusiasm. They work perfunctorily, although they do not neglect their obligations. These are disenchanted individuals who reduce their involvement and work without any passion because they find no meaning or amusement in their tasks they perform.

"The underchallenged teacher continues to do a professional job, does not especially resent the work, but does not especially look forward to it either. Teaching has lost its meaning..."

(Farber, 1991a, p. 95)

#### 2.2. Lack of personal development

Underchallenged subjects feel dissatisfaction on thinking that they are not developing as persons through their work. This is because they do not see their talents recognized in performing tasks that do not provide new challenges for them. Farber refers to this characteristic when he speaks of

*"Individuals whose range of talents are insufficiently recognized or exercised in their professional settings"*.

(Farber, 1990, p. 42)

Subjects of this type are focused on obtaining a kind of reward that does not seem to be reached in the performance of their tasks. They think their capacity and talent is above what is required of them by their job, and that they do not use their skills enough to identify themselves with it. In words of one patient, (Joan, twenty-six, primary teacher, four years' experience):

"I feel like I have outgrown my job...I know it sounds conceited, but I feel smarter than my job..."

(Farber, 1991a, p. 96)

They seem to be possessed by very demanding expectations with respect to the use of their abilities, which leads them to think that their current job only makes their personal development more difficult as it does not set them sufficient challenges. Farber describes Joan in this way:

"She came into therapy feeling that, given her abilities, she could or should be doing something more challenging and wondered why this wasn't the case."

(Farber, 2000b, p. 687)

These subjects have built up a narrowly-defined idea of their job and therefore find it totally lacking in interest. They have also lost their sense of proportion when considering their success at work and in other areas of their lives. They do not reach the point where their self-esteem is damaged. Although with an outlook that perhaps it will be in the future, their discontent leads them to question whether this field of work really is suitable for them.

"They have not incurred damage to their self-esteem... instead, they have begun to realistically sense that their self-esteem might well be damaged if they continue in work that they find unfulfilling and insufficiently demanding of their skills and abilities."

(Farber, 1991a, p. 94)

#### 2.3. Contemplating another job

The dissatisfaction experienced by these subjects leads them to contemplate other kinds of work, and to question the suitability of their current job, to the point where they weigh up the possibility of or desire other employment options. Individuals in this group seem to cope with disenchantment in their jobs by fantasizing over the possibility of taking on another more gratifying job. Subjects with this profile are invaded by feelings of doubt, restlessness and ambivalence towards their work, and propose new horizons for themselves in order to resolve them.

"...over time the underchallenged teacher begins to perform the work more perfunctorily, begins to question more whether this is the right field, begins to withdraw energy and enthusiasm."

(Farber, 1991a, pp. 94-95)

These ideas of giving up their profession could become affected by the appearance of guilt feelings, which partly attenuate the desire for change. This guilt may arise from their having lost the objective view of their natural entitlement to pursue their own needs. Nevertheless, these individuals will develop justifications and reasoning to explain their situation, either in the case where they take the decision to remain in their job or when they end up leaving it for another. Commenting on the case of Jill (thirty-eight, primary school teacher, seven years' experience), Farber says

"She felt somewhat guilty leaving teaching (to go into public relations) but justified it by reminding herself that she had given four good years to teaching and that she had certainly done "her share" of public service."

(Farber, 1991a, p. 97)

#### 2.4. Monotony and boredom

The prevailing detachment and lack of personal development in this profile is accompanied by a type of distress caused by boredom and the lack of stimulus, the source of which could be related to subjects performing tasks perfunctorily. Farber thinks that

"This is the group who feel stuck doing the same things every year and who, as a result, feel moribund, stale, left behind."

(Farber, 1991b, p. 122)

Repetitive and detached performing of functions, as if on an assembly line, doing the same thing over and over, day after day and year after year, will give rise to a stressful work atmosphere caused by routine and monotony. In these conditions, the underchallenged subject seems to feel trapped in his or her job. Joan expressed this thus,

"I am doing the same things over and over again... I just do not feel like doing it anymore..."

(Farber, 1991a, p. 96)

#### 2.5. Absence of overload-induced stress

Underchallenged subjects do not seem to have to cope with large amounts of work, and are consequently not excessively fatigued or suffer as a result of it. In Farber's words,

"...underchallenged" subtype of burnout, wherein an individual is faced not with an excessive degree of stress per se (i.e. work overload)..."

(Farber, 2000b, p. 677)

Nor do they perceive many difficulties in performing their tasks properly, so they are seen to be free from this type of anxiety and can perform their tasks with relative ease. They feel that they have problems at work relatively well under control and do not feel worn out by unwanted obstacles; nor do they become overwhelmed or angry because of them. As indicated by Farber,

"(This) type of burned out individual is neither fired up by unwanted obstacles, nor weighted down and overwhelmed by them."

(Farber, 1990, p. 40)

The attitude of indifference to work in jobs without major demands gives rise to a way of performing tasks without taking on too much stress. Here Farber refers to Jill; even after having left her job,

"She felt as if she had managed the strains of work relatively well and felt pleased at the job she had done."

(Farber, 1991b, p. 121)

### 3. Worn-out type

The worn-out profile consists of dispassionate subjects who have reduced their level of involvement to the point of neglecting their responsibilities. These are workers with a degree of pessimism that has led them to lose all enthusiasm for their job, and have chosen to give up any effort in the face of the setbacks experienced. In this respect they are,

"Those who in response to frustration give up entirely"

(Farber, 1990, p. 35).

#### 3.1. Neglecting responsibilities

The most relevant characteristic of the worn-out type is neglect. This can be understood as a lack of personal involvement in tasks until they respond to any difficulty by giving up. This idea is present in the corpus through segments such as that used to introduce this profile, or in the following,

"These worn out individuals are simply not as personally invested in their work"

(Farber, 1990, p. 40)

Worn-out workers are so aware of the difficulties that they reduce their sense of purpose to the point of managing to disconnect from their work. They play down the importance of tasks and minimize their objectives, with the feeling that they can no longer give of themselves. In the words of a patient, Jim (forty-one, high school teacher, ten years' experience),

"I know I get back less by giving less, but I just can't give anymore. I just don't give a damn."

(Farber, 2000b, p. 679)

Despite obtaining less personal gratification (achieving results that are not very flattering, in a job that is not very well done), these subjects reduce their level of involvement to the extreme of neglect as a way of balancing efforts and rewards. In this sense, Farber says that

"Worn-out teachers react to stress not by working harder but rather by working less hard; they attempt to balance the discrepancy between input and output by reducing their input."

(Farber, 1991a, p. 87)

They accept neglecting their responsibilities as a way of coping with difficulties, stress and frustration in a final attempt before seeing themselves affected by their work.

"worn-out workers have quit before they become totally consumed by their work."

(Farber, 1991a, p. 87)

#### 3.2. Absence of control over results

These subjects are worn out by the build-up of frustration brought about by having to cope with situations they feel they have no influence over. According to the author,

"They have been worn down by the cumulative effects of dealing with situations that they perceive as beyond their control..."

(Farber, 1991a, p. 87)

A condition that may favour the appearance and evolution of feelings of lack of control is when the worker continually has to deal with difficult-to-solve problems, especially if he or she has not come up with an adequate coping strategy. In these circumstances, worn-out subjects may even think they are immersed in a context plagued with hopeless situations, denying that their actions could have any effect on achieving better results.

"...he feels that several situations are 'out of control' and that nothing he does can make a difference..."

(Farber, 2000b, p. 678)

According to the theory of learned helplessness, subjects of this type may experience deterioration in the way they deal with situations owing to their lack of confidence. Within the framework of this theory, we can understand the lessening of motivation in these subjects as a consequence of the damage done to their expectations of control. In Jim's words,

"I just don't care that much anymore...I don't believe what I do or don't do makes much of a difference."

(Farber, 2000b, p. 678)

Worn out subjects are convinced the results will be disappointing, regardless of whatever they do, and that nothing they might try will be able to change their situation. Continued experience of difficult-to-handle situations, together with the inner feeling of having no control over outcomes, has damaged their perception of their effectiveness, and, in the end, their willingness to face them. Farber makes reference to Hal (fifty-six, high school teacher, thirty years' experience), a patient who did not get involved because he thought that

*"It is not worth it..."*.

(Farber, 2000b, p. 678)

Jim expresses his situation the following way:

"Even when I've tried my best, the successes have been less than overwhelming and God knows never appreciated."

(Farber, 1991a, p. 88)

#### 3.3. Problems with organization and reward system

The neglect characteristic of this profile may also be explained by a background of prior learning within an organization managed with bureaucratic rules and demands, with an organizational system that does not recognize effort and dedication, in conditions of low autonomy.

"...seems to be most often manifest among more experienced individuals working in institutions with particularly oppressive bureaucratic structures. These individuals have been worn down by organizational politics, by seemingly petty rules and demands, by low pay and low autonomy..."

(Farber, 1990, p. 42)

According to this view, workers with the greatest risk of suffering from this type of burnout are those working in large organizations who perform tasks under the subjective impression of having little support, and perhaps being guided by unrealistic expectations with regard to the possibilities of being shown gratitude and appreciation for their work. According to the author,

*"in settings offering little opportunity for advancement or recognition"*.

(Farber, 1990, p. 42)

#### 3.4. Difficulties in performing tasks

Worn-out subjects perceive the obstacles preventing them from doing effective work as oppressive, and they feel disappointed and discouraged when faced with difficulties that do not allow them to perform their tasks properly.

"Obstacles to effective work, therefore, are seen as oppressive by these individuals and tend to dampen (rather than heighten) their motivation."

(Farber, 1990, p. 40)

They feel overwhelmed by the structure that imposes an excessively narrow definition of what can be expected of their performance, based on general and binary appraisals (everything is wrong), instead of specific and flexible ones (reasonable progress has been made in this case). Subjects of this type focus on negative aspects and feel frustrated with their working conditions, owing both to lack of resources (personal and/or material) and to an excessive workload. They are therefore willing to recognize situations that pose some sort of difficulty as failures. Farber says that,

"...the bottom line is their willingness to face the fact that they cannot achieve the goals they had once set for themselves..."

(Farber, 1991a, p. 89)

They seek more comfortable positions and stop worrying about things. They reason their failures and devise complaints through which they can attribute blame to external factors. They feel that nobody understands how difficult it is to do their work well and that nobody understands what they have to put up with. They surround themselves with people who share the same outlook on things. The author quotes Shanker in saying that,

*"...their beef is with the system and circumstances that constantly impede the realization of their goals"*.

(Farber, 1991b, p. 123)

#### 3.5. Depressive symptomatology

Subjects of this type suffer from emotional exhaustion to the extent that, according to Farber, they may develop burnout together with depressive symptoms.

"The worn-out teacher manifests symptoms akin to those of depression, including a perceived loss of self-esteem, and often requires cognitive approaches that aim to rebalance his or her perceptions."

(Farber, 2000b, p. 677)

As with subjects suffering from depression, worn-out workers have damaged self-esteem. Moreover, the pessimism they are imbued with leads them to make errors of judgement when interpreting present events and perceiving the future.

*"...tend to minimize successes, maximize failures, and perceive the future as inevitably as bleak as the present"*.

(Farber, 2000b, p. 680)

They cope with daily challenges and difficulties with apathy and lack of energy, and feel worn out and fatigued, which reduces their involvement in their work without taking the quality of their service into account.

"Those who are worn out have incurred damage to their sense of self-esteem -they are no longer personally invested in performing well on the job."

(Farber, 1991a, p. 89)

Workers of this type experience feelings of helplessness, desperation, discouragement, irritability and guilt. Hal, who was treated by the author and who finally gave up the profession, expressed the following opinion:

"I feel guilty sometimes about the good kids I am not teaching as well as I should..."

(Farber, 1991b, p. 123)

### b) Conceptual Characterization Of The Model

In this section we give a summary of the properties of the different types, with the aim of providing a parsimonious presentation of the proposal until we are left with a single category that gives meaning to the differentiation established in the profiles.

### 1. Frenetic type

The frenetic type profile can be briefly characterized by the following properties: "involvement", as an increasingly greater effort to face the difficulties of work, in an attempt to raise the probability of producing expected results; "ambition", in the sense of a considerable need for achievements and external approval resulting from brilliant operations; "rejection of failure", as an absence of acknowledgement of failure or of one's own limitations in the belief that results reflect personal worth; "overload", in the sense of risking one's health and personal life for work, investing intense and uninterrupted effort; and "anxiety and irritability", in excessive worry with work demands, until one feels overwhelmed and has difficulties relaxing or sleeping.

The properties of "ambition" and "rejection of failure" appear to be closely related. A considerable need for achievements and external approval could determine the absence of acknowledgement of failure and of one's own limitations. Therefore, we will now refer to both as a single term, "grandiosity". On the other hand, the property of "anxiety and irritability" gives the impression of sharing symptoms of anxiety disorders too closely, so we chose to eliminate it. Thus, we have three subcategories to describe the frenetic type: "involvement", as an increasingly greater effort to face the difficulties of work; "grandiosity", in the sense of a considerable need for achievements, together with rejection of failure or limitations; and "overload" which refers to putting one's health and personal life at risk for work.

### 2. Underchallenged type

The underchallenged type presents: "indifference", as a way of performing work in a superficial and detached manner, although without neglecting all responsibilities; "lack of development", defined as dissatisfaction felt on not seeing one's talents acknowledged in the performance of tasks that pose no new challenges; "contemplating another job", in the sense of questioning the suitability of one's current job and weighing up other employment options; "boredom", which may be seen as one's experience of work as routine and monotonous, owing to the perfunctory performance of tasks; and "absence of overload-induced stress", which corresponds to a way of performing tasks without too much stress as there is no need to cope with major demands.

The properties "lack of development" and "contemplating another job" can be considered closely related. The fact that one does not develop at work could be significant when it comes to desiring other employment options. We therefore will refer to both properties simultaneously as "lack of development", in the understanding that this is the determinative property. The "absence of overload-induced stress" could be associated with "boredom" given that both appear to refer to a monotonous environment produced by lack of stimulus. We will therefore give the name "boredom" to the property combining both characteristics. Thus, we have three subcategories to characterize this profile: "indifference", as the way of performing tasks in a superficial and detached manner; "lack of development", owing to the dissatisfaction of not seeing one's talents acknowledged until other employment options are contemplated; and "boredom", in the sense of monotony, owing to the perfunctory performance of tasks without experiencing stress or major demands.

### 3. Worn-out type

Worn-out workers present: "neglect", as a lack of personal involvement in work-related tasks, leading one to give up as a response to any difficulty; "lack of control", as the presence of feelings of desperation caused by absence of control over results; "lack of acknowledgement", when one feels the organization he or she works for does not acknowledge efforts and dedication; "difficulties", as a feeling of oppression owing to the lack of resources and difficulties preventing one from performing effective work; and "depression", as the presence of depressive symptomatology.

The fact that one feels great oppression brought about by the "difficulties" faced in performing tasks, given that they prevent them from being carried out satisfactorily, could be related to the feelings of desperation caused by "lack of control". We have therefore decided to group both properties into one, which we now call "lack of control". We have also eliminated depressive symptomatology as it is more characteristic of other types of emotional disorders. In short, we can characterize the worn-out type through of the following subcategories: "neglect", as the lack of involvement in work tasks to the point of giving up in the face of any difficulty; "lack of acknowledgement", as the feeling of not seeing one's efforts and dedication recognized; and "lack of control", as the desperation caused by absence of control over results when experiencing difficulties in performing tasks.

### 4. Core category: degree of dedication at work

**Figure 1:**
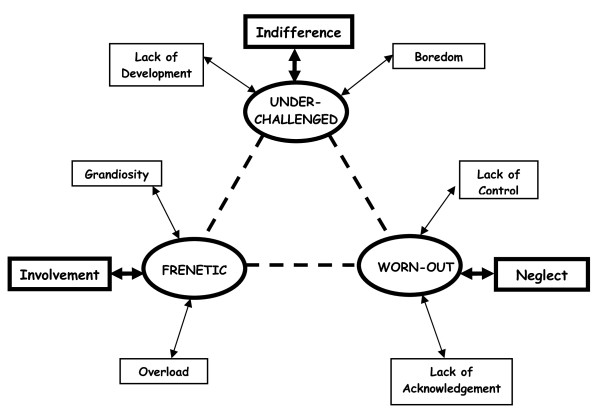
**Graphic representation of the conceptual characterization of the model**.

Figure 1 allows the properties defining each of the clinical profiles to be appreciated. We have highlighted the characteristics of involvement, indifference and neglect as being the values making up the core category of the classification, the category capable of fitting the entire classification together. This category is based on the degree of "dedication" at work. The involvement and neglect values corresponding to the frenetic and worn-out types, respectively, appear as opposites so they have been placed on opposite sides in the new dimension. On the other hand, the place taken by the indifference value of the underchallenged profile is not exactly clear.

Looking once more at the corpus, we can however see that on one occasion, the author of the classification described this type as

*"...those who are relatively immune to frustration -who neither work harder nor give up but instead perform their work perfunctorily, having lost interest in work they now find unchallenging and unstimulating"*.

(Farber, 1990, p. 40)

This description of the underchallenged type as the negation of the basic properties characterizing the other two provides a clue on how to approach the matter of the formal establishment of the classification criterion. This aspect is dealt with in the following section.

### c) Structural Definition Of The Classification Criterion

**Figure 2:**
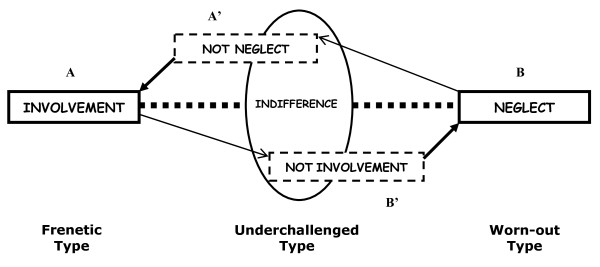
**Qualitative burnout typology according to the 'dedication' classification**.

The complete set of properties in the proposal seems to be arranged around the core category of "dedication", the end values of which are involvement in work, on the one hand, and neglect of tasks, on the other. These are two basic strategies for coping with difficulty - the involvement strategy, as increasingly greater effort when face with frustration, and the neglect strategy, in the sense of reaching the point of giving up when faced with any difficulty. In this regard, we now go on to confirm whether the core category can in fact logically group all of its values (involvement, indifference and neglect) by means of a single dimension. For this purpose, we split the two end-terms and negated each of them to come up with the four terms that would give substance to the semiotic square. Figure 2 shows the network of relations in which the semantic microuniverse is arranged represented by this category, recognizing the positions of virtual meaning defined by the network by means of the relations of contrariness (A-B; A'-B'), contradiction (A-B'; B-A') and the ability to be complementary (A'-A; B'-B).

According to the earlier elementary relations of meaning, the logically possible values of the complete systematic typology [26] will be: 1- workers who become involved in their jobs (A), i.e. who invest greater effort when faced with difficulties; 2- those who do not neglect their efforts (A'), who do not give up when faced with any obstacle; 3- those who do not become involved (B'), or do not invest greater effort when face with frustration; and 4- those who neglect (B), in the sense of giving up when faced with any problem.

From a logical perspective, these are the possibilities created by the results of the semiotic square over the primitive core category end-terms. However, in order to adapt it to the original profiles of the preliminary proposal, the four possible solutions must be simplified to three. We therefore accept a partial correspondence between the sub-contrary terms (A'-B') and reduce the terms "not involvement" and "not neglect" to one, corresponding to the value of "indifference". This means that indifference is defined as the absence of involvement and absence of neglect at the same time (Figure 2), i.e. not investing great effort but without neglecting tasks, which is consistent with the description pointed out by the author in the previous section.

When using the intersection between the negations of the primitive end-terms as the intermediate position for locating the indifference characteristic, the semantic axis of "dedication" appears as a dimension that allows the formal articulation of all the values of the core category, which then becomes the new classification criterion for the typology, now systematized through a system of relations.

By means of the earlier semantic analysis, we can appreciate the theoretical core underlying the classification of the preliminary typology, over which the sets of meaning devised by the author are constructed. This discovery will enable us to propose very brief definitions of the initially-proposed clinical profiles. These definitions will be based on the attitudes subjects take compared to the feelings of insignificance burnout arouses, according to the degree of "dedication", as a way of coping with work-related problems and frustrations.

The resulting definitions of this entire process are: a) frenetic type, copes with work-related difficulties with greater involvement in tasks and invests increasing effort; b) underchallenged type, copes with work superficially through indifference and detachment, without too much involvement although without neglect; c) worn-out type, copes with work-related difficulties by neglecting responsibilities, in the sense of lack of involvement in work to the point of giving up when face with any difficulty.

## Discussion

**Figure 3:**
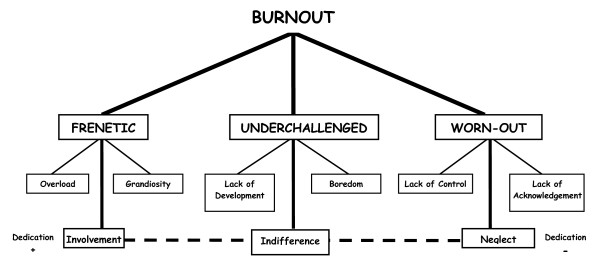
**Structure of the systematized typology**.

The model described to this point allows differences to be established between those affected by burnout syndrome. The degree of "dedication" to work dimension becomes the classification criterion to which the remaining properties are connected. This facilitates the recognition of the three clinical profiles (Figure 3 and Table 1).

A recent study [27] expressed the relation between work overload and psychological distress, emotional fatigue and depersonalization. According to the results of the study, the difficulties in balancing work and family demands are a significant source of stress, which is congruent with our definition of overload. This study also highlights participating workers as having extraordinary levels of personal performance, with the aim of keeping a level of work satisfaction according to an internalized ideal model. Consequently, frenetic type grandiosity could be associated with a professional identity based on great expectations (perhaps also within a work organization with strong demands), which would compel workers to greater "acceleration". This acceleration or higher involvement could be related to the "meaning in the workplace" of Borritz et al. [28], which consistently predicts burnout syndrome. It can be said that "in order to burn out, one first has to be alight", or in other words, the most committed subjects have a higher likelihood of developing the condition, a process that begins when work ceases to have meaning [5].

Lack of development could be related to dissatisfaction with work, proposed as a cause of burnout in the model by Janssen et al. [29]. Along the same lines, the study by Borritz et al. [28] concludes that low perception of opportunities for personal development in a job is a predictor for burnout in three years. Another study [30] made the observation that thinking there could be other jobs that better acknowledged one's capacity was related to the causes of the syndrome and with burnout itself. The same study expressed that lack of gratification and monotony in tasks was associated with both. In this regard, boredom and apathy have been related to the absence of personal and professional development, and it is thought that job rotation could diminish this [31]. The syndrome development model proposed by Moreno et al. [32], confirmed in structure by Montero et al. [33], considers monotony, detachment and low identification with work as cases of burnout. In both the procedural model by Moreno et al. [32] and the study by Dickinson and Wright [31], indifference at work appears as detachment as a way of performing tasks superficially.

Desperation caused by low predictability could correlate with burnout levels [28]. Lack of control could also be associated with situations of low authority in decision-making, which has been related to emotional exhaustion [34]. In this respect, attributions with external locus of control have been related to high levels of emotional fatigue and depersonalization [35]. Lack of acknowledgement appears to be related to low satisfaction with work, a feeling that may influence development of the syndrome [36]. The latter work expresses how job conditions, such as low pay or large administrative workloads, diminish job satisfaction. With regard to job neglect, burnout is a predictor for illness-related absences from work [37]. Job satisfaction levels appear to be related to stress, burnout and abandoning careers [38]. Apathy at work could be related to ineffectiveness [39], and inversely to drive, participation and absorption, which characterize the opposite of burnout [40].

However, this interpretive framework is not without limitations. A number of the author's descriptions can be found which do not exactly fit the configuration of characteristics in the proposed model. For example, feelings of desperation caused by lack of control can be gauged from an isolated quote taken from a patient (Susan, thirty, high school teacher, three years' experience) classified by the author as frenetic.

"I really feel like I'm at the edge...I'm working unbelievably hard and I'm not sure It's getting better...I'm not sure how much longer I can do this."

(Farber, 2000b, p. 683)

Farber also comments on a frenetic teacher (Paula, twenty-six, primary teacher, two years' experience), who chose to give up her job when she felt she could not reach her objectives.

"She felt she could not control the students in her class, could not round up enough books for the slower students, and could not find enough time or energy to make use of the support that some colleagues were offering."

(Farber, 1991b, p. 119)

It is possible to find isolated descriptions of the underchallenged type in which the author points out certain feelings of lack of recognition.

"Here the stresses of work are not great but neither are the rewards -particularly those of a psychological nature."

(Farber, 1990, p. 42)

Or the case of teachers classified by Farber as underchallenged (for example the case of Jill, thirty-eight, primary school teacher, seven years' experience), who chose to change job in search of greater remuneration for their intelligence and ability.

"...this is the group who leave not to escape from too much stress but to find greater sources of stimulation -and often greater remuneration for their intelligence and ability."

(Farber, 1991b, p. 122)

What is certain is that the author explicitly acknowledges these inconsistencies and points out that besides the described types, there are profiles that defy classification because they are a cumulus of the other three, because the set of characteristics do not coincide with any of the proposed types or even because he came across professionals that oscillated between the three categories [8]. Likewise, the author of the preliminary classification gives cause (although not explicitly and with the limitation of not being able to integrate the profile underchallenged) to understand the typology proposal as evolving over time. For example, with reference to the worn-out type, he says,

"it is possible that those teachers who now appear burned out were once the most dedicated teachers in their schools."

(Farber, 1991a, p. 89)

This new element of analysis raises the possibility of interpreting the typology from a longitudinal perspective, understanding burnout as a process involving diminishing dedication to work, which ends in neglect and breaking of the commitment. This proposal agrees with the position taken by Schaufeli, Salanova et al.[40] regarding engagement being the opposite of burnout. Development of the syndrome may be seen as a gradual process of commitment erosion. The demands/resources model by Schaufeli and Bakker [41], revised and expanded by Lorente et al. [42], expresses the role of quantitative overload as a cause for exhaustion and, ultimately, of dedication. The progressive diminishing of involvement in work could reduce gratification or professional recognition, and undermine feelings of self-efficacy, ending with the neglect of responsibilities that is characteristic of the worn-out type. This is in line with Bandura's theory [43,44] according to which self-efficacy is a predictor of persistence or abandonment in the face of obstacles and difficulties.

## Conclusion

Understanding the development of burnout syndrome in this way, as a succession of stages characterized by the progressive diminishing of dedication to work, could serve, not only for the establishment of specific therapies according to the presented profile, but also to clarify the dimensions of the proposed factors when it comes to expanding the study of burnout towards the opposite, positive aspects of the syndrome (drive, participation and absorption), the source of so much controversy [45].

Studies reviewing the efficacy of treatment and prevention interventions in workers with burnout are not too optimistic [46-48]. Limited evidence is available for a small reduction in stress levels from person-directed, person-work interface, and organizational interventions among health care workers. It is probable that encouraging the positive side of the burnout model we have proposed could be of great interest in the prevention of the syndrome. Person and organizational interventions aimed to improve drive, participation and absorption could be more effective that traditional cognitive therapy-based programs, because they focus on the core concept of burnout. Nevertheless, these questions would have to be resolved through future empirical research, given that they are beyond the scope of this work.

## Competing interests

The authors declare that they have no competing interests.

## Authors' contributions

JM and JGC conceived the research study. JM, DMM and YLdH carried out the content analysis. All authors interpreted the results, drafted the manuscript and read and approved the final manuscript.
